# Response to “Understanding chronic Covid-19”

**DOI:** 10.1016/j.eclinm.2022.101551

**Published:** 2022-07-10

**Authors:** Adam Hampshire, David K. Menon

**Affiliations:** a Department of Brain Sciences, Imperial College London, UK; b Division of Anaesthesia, Department of Medicine, University of Cambridge, UK; c Cambridge University Hospitals National Health Service Foundation Trust, UK; d Wolfson Brain Imaging Centre, Department of Clinical Neurosciences, University of Cambridge, UK

We agree that cognitive dysfunction after COVID-19 is likely mediated by indirect mechanisms. A possible mechanism we are currently exploring is autoinflammatory/autoimmune injury, evidenced by elevated brain-injury biomarkers that scale with systemic illness. Similar elevations occur in intensive care unit (ICU) patients with severe influenza. Lasting cognitive problems after COVID-19 occur following other critical illnesses.^1^ Therefore, indirect mechanisms have broad relevance.

Our supplemental analyses suggested that patients without mechanical ventilation also had significant cognitive deficits. Associations between our computerised tests and diverse conditions including depression, anxiety, insomnia, diabetes, cardiovascular disease, autoimmune disorders are of negligible-small scale,^2^ but could confer vulnerability to worse cognitive outcomes post COVID-19. The correlations of fatigue score with cognitive deficits and with gender were negligible in our study. Nonetheless, fatigue can become apparent with sustained task performance after even mild COVID-19^3^ and should be investigated further.

As noted in our discussion, caution is needed before inferring cause and effect from correlational analyses. This is also because people follow different cognitive developmental and decline trajectories through their lifespans, which may correlate with vulnerability to severe COVID-19. We can therefore suggest inference only by synthesising the body of available information. Objective, self-report, clinical and epidemiological evidence converge in indicating chronic cognitive deficits after severe COVID-19. Indeed, in agreement, a subsequent larger study than ours has reported broadly similar findings.^4^ Large-scale studies are required to understand the mechanisms that mediate the COVID-cognition association, identify treatment approaches, and determine how these findings generalise to other severe viral illnesses.

## Contributors

Drafting of the letter: A.H. & D.K.M.

## Declaration of interests

Professor Hampshire reports grants from UK Dementia Research Institute, grants from NIHR Imperial Biomedical Research Centre, and grants from NIHR, outside the submitted work; and is Co-director and owner of H2 Cognitive Designs Ltd and director and owner of Future Cognition Ltd, which support online cognitive studies and develop custom cognitive assessment software respectively. Professor Menon reports grants from Lantmannen AB, grants from GlaxoSmithKline Ltd, personal fees from Calico LLC, personal fees from GlaxoSmithKline Ltd, personal fees from Lantmannen AB, other from Integra Neurosciences, outside the submitted work; and reports leadership and fiduciary roles for Queens’ College, Cambridge, Intensive Care National Audit and Research Centre, London, and European Brain Injury Consortium.
